# Cross-sectional survey on cancer patients’ concerns and consultation needs with pharmacists at the time of initial diagnosis

**DOI:** 10.1186/s40780-025-00467-w

**Published:** 2025-07-10

**Authors:** Tomofumi Watanabe, Atsunobu Sagara, Tomoya Abe, Masato Komuro, Koharu Yubune, Satoshi Yoshikawa, Hiroyuki Terakado

**Affiliations:** 1 Department of Clinical Epidemiology, School of Pharmacy and Pharmaceutical Sciences, Hoshi University, 2-4-41 Ebara, Shinagawa-ku, Tokyo Japan; 2 Department of Pharmacy, Japanese Red Cross Medical Center, 4–1-22 Hiroo, Shibuya-ku, Tokyo Japan; 3 Department of Pharmacy, Saitama Cancer Center, 780 Komuro, Ina-machi, Kitaadachi-gun, Saitama, Japan; 4 Department of Medical Informatics, Bureau of System Infrastructure Development, Japan Institute for Health Security, 1–21-1 Toyama, Shinjuku-ku, Tokyo Japan; 5 Section of Informatics Platform for Medical Research and Collaboration, Japan Health Research Promotion Bureau, 1–21-1 Toyama, Shinjuku-ku, Tokyo Japan; 6 Department of Pharmacy, Japan Institute for Health Security, National Center for Global Health and Medicine, 1–21-1 Toyama, Shinjuku-ku, Tokyo Japan; 7 Faculty of Pharmacy, Iryo Sosei University, 5–5-1 Chuo-dai Iino, Iwaki, Fukushima Japan

**Keywords:** Oncology pharmacy, Cancer diagnosis, Pharmacist consultation, Pharmaceutical care, Hospital pharmacist, Community

## Abstract

**Background:**

The immediate post-diagnosis period is a critical phase for cancer patients, marked by significant informational and emotional distress. Although pharmacists are well-positioned to provide support during this time, limited research has investigated patients’ specific concerns and consultation needs immediately after diagnosis, particularly in differentiating between hospital and community pharmacists. This study aimed to clarify cancer patients' concrete concerns and consultation preferences immediately following diagnosis, with a focus on the respective roles of hospital and community pharmacists.

**Methods:**

A nationwide cross-sectional web-based survey was conducted among 1,031 adult cancer patients in Japan. Participants selected relevant concerns from a 21-item structured questionnaire across four domains: Cancer and Cancer Treatment (CCT), Cancer Pain and Palliative Care (CPPC), Medications Other Than Cancer Treatment (MOCT), and Daily Life During Cancer Treatment (DLCT). For each concern, participants indicated whether they preferred to consult hospital pharmacists, community pharmacists, or both. McNemar tests were used to compare paired proportions (*P* < 0.001).

**Results:**

A total of 89.2% of participants reported at least one concern at diagnosis. The most frequently reported concerns were treatment-related, including side effects (49.2%), treatment costs (48.0%), psychological distress (41.6%), and mechanisms of anticancer drugs (38.8%). Patients expressed significantly stronger preferences for consulting hospital pharmacists over community pharmacists on treatment-specific topics such as side effects (34.7% vs. 13.8%), drug mechanisms (39.3% vs. 18.5%), and medications to relieve physical discomfort (36.1% vs. 17.0%) [all *P* < 0.001]. In contrast, MOCT-related concerns, such as drug interactions and medication management, elicited similarly high consultation preferences for both pharmacist types (> 40%). DLCT and CPPC-related concerns were associated with relatively lower consultation demands overall.

**Conclusions:**

Cancer patients experience diverse and substantial informational and emotional needs immediately after diagnosis. Hospital pharmacists are particularly valued for treatment-specific support, while both hospital and community pharmacists are seen as essential resources for broader medication-related concerns. Enhancing cooperation between hospital and community pharmacists, and strengthening pharmacist-led support tailored to patients' needs at diagnosis may significantly improve patient-centered care and quality of life.

## Background

Cancer remains the leading cause of death worldwide [1], and receiving a cancer diagnosis imposes a significant physical and psychological burden on patients [2, 3]. In particular, the period immediately following diagnosis is not merely a pre-treatment phase but represents a uniquely vulnerable and decisive moment in a patient’s life [4]. During this time, patients often experience overwhelming psychological distress due to prognostic uncertainty, confusion about treatment options, disruption of livelihood, and financial anxiety—all occurring simultaneously [5, 6]. In such a critical situation, the presence or absence of timely and appropriate support from pharmacists may substantially influence subsequent treatment behaviors and medication adherence [7–10].

However, most previous studies have focused on the middle or terminal stages of cancer treatment, and only a few have systematically investigated patient needs during the “critical window for supportive intervention” immediately after diagnosis [11–14]. Notably, no published studies—either in Japan or internationally—have directly compared patients’ consultation preferences for hospital pharmacists versus community pharmacists using a consistent set of indicators.

With the advancement of outpatient cancer pharmacotherapy and growing emphasis on community-based care, pharmacists are increasingly expected to provide holistic support that goes beyond conventional drug therapy and addresses patients’ psychosocial concerns as well [15, 16]. Particularly in the early stages following diagnosis, it is crucial to establish a support framework that can promptly and effectively respond to patients’ questions and anxieties before this limited window of opportunity closes.

This study aimed to clarify the specific concerns patients face immediately after a cancer diagnosis and directly compare their consultation needs regarding hospital and community pharmacists using a unified set of indicators. By identifying the types of support expected from each group, the study seeks to highlight key issues for enhancing the professional role of pharmacists in cancer care.

## Methods

### Survey period and participants

The survey was conducted over 7 days from January 23 to January 29, 2025. Participants were recruited from the panel maintained by Macromill, Inc. (Tokyo, Japan), a marketing research company. As of January 2025, the company's registry included approximately 36 million monitors. A total of 22,419 individuals aged 17 years or older who had been diagnosed with cancer and had received treatment in Japan were invited to participate in the web-based questionnaire survey. Ultimately, 1,031 valid responses were obtained.

To prevent fraudulent responses, Macromill implements two quality control measures: a trap question survey conducted every 6 months, and mandatory annual updates of monitor registration information [17, 18].

The required sample size was estimated to be approximately 400, assuming a 50% response distribution, 5% margin of error, and 95% confidence level. The number of valid responses obtained (*n* = 1,031) exceeded this threshold.

The questionnaire included items categorized into four areas, reflecting concerns that pharmacists may assist with during cancer treatment. These items were refined based on feedback from public health professionals and family members of cancer patients in a previous study.

### Questionnaire items

The questionnaire used in this study was partially modified from the item titled “Concerns and Problems of Cancer Patients that Pharmacists Can Support” (Table 1), which was originally included in a previous study, “Survey on the Role of Hospital Pharmacists in Outpatient Cancer Treatment,” conducted by the Department of Pharmacy at Kyoto University Hospital in September 2023 [19]. Notably, the items in the original questionnaire had already been refined prior to that study, based on feedback from public health professionals and family members of cancer patients.

**Table 1:** Cancer patients’ concerns that pharmacists can support

Cancer and cancer treatment (CCT)
□ The mechanism of action, efficacy, and effects of anticancer drugs
□ Side effects associated with cancer treatment
□ Medications to alleviate physical discomfort (side effects) caused by cancer or its treatment
□ The cost of cancer treatment
□ Mental distress (anxiety, worries, etc.) caused by cancer or its treatment (mental health care)
□ New cancer treatment methods (clinical trials, etc.)
Cancer pain and palliative care (CPPC)
□ Cancer pain (palliative care)
□ Medical opioids used for pain relief
□ Home care or hospice care
Medications other than cancer treatment (MOCT)
□ Regular medications not related to cancer treatment
□ Difficulty taking medications (tablets, capsules, powders, etc.)
□ Adjusting leftover medications that you cannot take (consultations about reducing medications)
□ Over-the-counter (OTC) medications
□ Health foods and supplements
□ Drug interactions
□ Correct medication usage (e.g., preventing missed doses)
Daily life during cancer treatment (DLCT)
□ Precautions and lifestyle habits during daily life (e.g., driving)
□ Work or studies during cancer treatment
□ Nutrition and diet
□ The impact of cancer treatment on pregnancy and childbirth
□ Changes in appearance (hair loss, skin disorders, etc.)

The classification used in the previous study—Cancer and Cancer Treatment (CCT), Medications Other Than Cancer Treatment (MOCT), and Daily Life During Cancer Treatment (DLCT)—served as the basis [19]. In this study, to more comprehensively capture patients’ concerns, items related to cancer pain and palliative care were reclassified from CCT and added as a new domain, Cancer Pain and Palliative Care (CPPC).

The questionnaire consisted of two main questions:

1. Whether the respondent had any concerns at the time of initial cancer diagnosis (multiple responses allowed).
2. For each selected concern, whether the respondent wished to consult a hospital pharmacist or a community pharmacist (yes/no).

In this study, the term “hospital pharmacist” refers to pharmacists working at medical institutions that provide cancer treatment, without differentiating between inpatient and outpatient settings. The aim was to assess patients’ consultation preferences for hospital pharmacists involved in cancer care as a unified professional group.

### Statistical analysis

Descriptive statistics were used to summarize respondent characteristics, including age group, sex, and cancer type. To compare consultation preferences between hospital and community pharmacists, the McNemar test was applied to paired binary data. When the total number of discordant pairs was < 25 or when the frequency of any cell was < 5, the mid-P method was used to enhance the accuracy of the analysis.

Descriptive statistics were calculated using Microsoft Excel (Microsoft Corp., Redmond, WA, USA), while the McNemar test and mid-P method were performed using JMP Pro 18 (SAS Institute Inc., Cary, NC, USA). Given the large sample size and high statistical power of this study, the significance level was conservatively set at *P* < 0.001 to reduce the risk of identifying trivial differences as statistically significant.

## Results

### Background of the study population

Age distribution, sex, and cancer types of the respondents are presented in Table 2. Of the participants, 62.1% were male and 37.9% were female. The most common age group was 60–69 years (33.1%), followed by 70–79 years (23.7%), and 50–59 years (22.8%), indicating that 83.4% of respondents were 50 years or older. Breast and colorectal cancer were the most frequently reported cancer types, accounting for 20.0% of the responses, followed by stomach cancer (16.0%), prostate cancer (13.0%), and lung cancer (12.0%).

**Table 2:** Background of the Study Population

Variable	n	(%)
Gender		
Male	640	(62.1)
Female	391	(37.9)
Age (years)		
15-39	48	(4.7)
40-49	124	(12.0)
50-59	235	(22.8)
60-69	341	(33.1)
70-79	244	(23.7)
** ≥ **80	39	(4.7)
Cancer type		
Breast cancer	206	(20.0)
Colorectal cancer (colon/rectum)	206	(20.0)
Stomach cancer	165	(16.0)
Prostate cancer	134	(13.0)
Lung cancer	124	(12.0)
Malignant lymphoma, leukemia, or multiple myeloma	44	(4.3)
Cervical/Endometrial/Ovarian cancer	37	(3.6)
Thyroid cancer	30	(2.9)
Bladder cancer	22	(2.1)
Oral/Pharyngeal/Laryngeal cancer	21	(2.0)
Esophageal cancer	16	(1.6)
Liver cancer	16	(1.6)
Pancreatic cancer	10	(1.0)

Values are expressed as n (%)

### Concerns at the time of initial cancer diagnosis

**Fig. 1:**
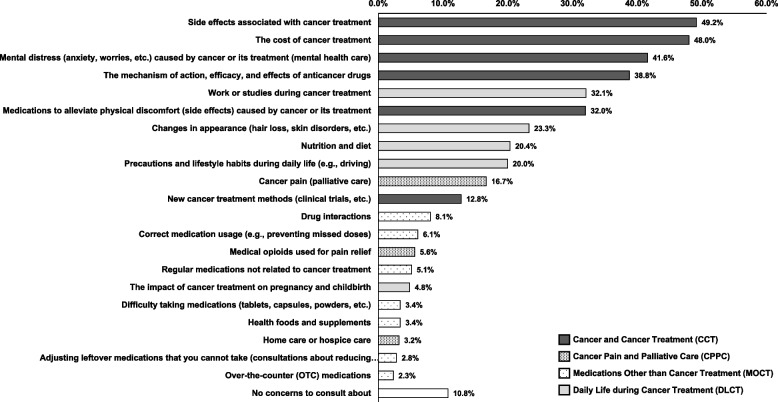
Distribution of concerns among cancer patients at initial diagnosis

Figure 1 shows the response rates for concerns reported by patients at the time of their initial cancer diagnosis. Overall, 89.2% of the respondents indicated that they had some type of concern or burden.

The items classified under CCT had the highest response rates. The most frequently selected concerns were “Side effects associated with cancer treatment” (49.2%), “Cost of cancer treatment” (48.0%), “Psychological distress (e.g., anxiety and worries related to cancer and its treatment)” (41.6%), “Mechanisms, indications, and effects of anticancer drugs” (38.8%), and “Medications to relieve physical discomfort” (32.0%). In contrast, “New cancer treatments (e.g., clinical trials)” was selected by only 12.8%.

This was followed by items related to DLCT, with relatively high selection rates for “Work or academic responsibilities” (32.1%), “Changes in appearance” (23.3%), “Nutrition and diet” (20.4%), and “Precautions in daily life” (20.0%).

Among items classified under CPPC, “Cancer pain” was selected by 16.7% of respondents, whereas “Use of medical opioids” (5.6%) and “Home care or hospice” (3.2%) were selected less frequently. Items under MOCT all had response rates of < 10%, including “Drug interactions” (8.1%), “Correct use of medications” (6.1%), and “Regular medications not related to cancer treatment” (5.1%).

### Pharmacist consultation intentions at the time of cancer diagnosis

Table 3 shows the proportion of respondents who expressed a desire to consult hospital and community pharmacists about their concerns at the time of initial cancer diagnosis, along with the comparative results between the two groups.

**Table 3:** Patient’s consultation intentions for hospital and community pharmacists

	Concerns and Problems of Cancer Patients that Pharmacists Can Support	N	Hospital pharmacist		Community pharmacist		*p *value
			n	(%)	n	(%)	
CCT	Side effects associated with cancer treatment	507	176	(34.7)	70	(13.8)	< 0.001
	The cost of cancer treatment	495	53	(10.7)	37	(7.5)	0.027
	Mental distress (anxiety, worries, etc.) caused by cancer or its treatment (mental health care)	429	66	(15.4)	41	(9.6)	< 0.001
	The mechanism of action, efficacy, and effects of anticancer drugs	400	157	(39.3)	74	(18.5)	< 0.001
	Medications to alleviate physical discomfort (side effects) caused by cancer or its treatment	330	119	(36.1)	56	(17.0)	< 0.001
	New cancer treatment methods (clinical trials, etc.)	132	30	(22.7)	13	(9.8)	< 0.001
DLCT	Work or studies during cancer treatment	331	39	(11.8)	25	(7.6)	0.004
	Changes in appearance (hair loss, skin disorders, etc.)	240	46	(19.2)	36	(15.0)	0.035
	Nutrition and diet	210	55	(26.2)	39	(18.6)	0.014
	Precautions and lifestyle habits during daily life (e.g., driving)	206	53	(25.7)	42	(20.4)	0.063
	The impact of cancer treatment on pregnancy and childbirth	50	14	(28.0)	11	(22.0)	0.022
CPPC	Cancer pain (palliative care)	172	46	(26.7)	30	(17.4)	0.002
	Medical opioids used for pain relief	58	26	(44.8)	16	(27.6)	0.019
	Home care or hospice care	33	10	(30.3)	9	(27.3)	0.688
MOCT	Drug interactions	83	48	(57.8)	50	(60.2)	0.715
	Correct medication usage (e.g., preventing missed doses)	63	40	(63.5)	33	(52.4)	0.178
	Regular medications not related to cancer treatment	53	34	(64.2)	26	(49.1)	0.049
	Difficulty taking medications (tablets, capsules, powders, etc.)	35	23	(65.7)	20	(57.1)	0.344
	Health foods and supplements	35	18	(51.4)	19	(54.3)	0.774
	Adjusting leftover medications that you cannot take (consultations about reducing medications)	29	20	(69.0)	14	(48.3)	0.065
	Over-the-counter (OTC) medications	24	15	(62.5)	11	(45.8)	0.227

Values are expressed as n (%). Statistical comparisons were performed using the McNemar test. *p* values<0.001 were considered statistically significant

*Abbreviations*: *CCT* Cancer and Cancer Treatment, *CPPC* Cancer Pain and Palliative Care, *DLCT* Daily Life during Cancer Treatment, *MOCT* Medications Other Than Cancer Treatment

The need for consultation with hospital pharmacists was significantly higher than that with community pharmacists for five of the six items classified as CCT, except “The cost of cancer treatment” (*P* < 0.001). In particular, “Side effects associated with cancer treatment” (34.7% vs. 13.8%), “The mechanism of action, efficacy, and effects of anticancer drugs” (39.3% vs. 18.5%), and “Medications to alleviate physical discomfort caused by cancer or its treatment” (36.1% vs. 17.0%). The need for consultation with a hospital pharmacist accounted for > 30% of the total responses, which was significantly higher than that for the other items.

In contrast, for all items classified under DLCT, the percentage of respondents wishing to consult hospital pharmacists was < 30%, and there were no statistically significant differences between hospital and community pharmacists. In contrast, for all items classified under DLCT, the percentage of respondents wishing to consult hospital pharmacists was < 30%, and there were no statistically significant differences between hospital and community pharmacists.

Although no significant differences were observed for MOCT items, > 40% of the respondents expressed a desire to consult both hospital and community pharmacists for all MOCT-related concerns, indicating a generally high level of need for consultation in this category.

## Discussion

### Statement of key findings

This study revealed that nearly 90% of cancer patients experienced some form of distress during the highly burdensome psychological period immediately after diagnosis. Concerns categorized under CCT were the most prevalent, particularly reflecting a strong need for specific information on cancer pharmacotherapy and a high demand for consultation with hospital pharmacists. On the other hand, although concerns categorized under MOCT were less frequently selected overall, consultation needs exceeded 40% for both hospital pharmacists and community pharmacists, indicating that pharmacists are expected to provide support for pharmacological management other than cancer. These findings suggest that hospital and community pharmacists play complementary roles in addressing patients’ diverse concerns at the time of diagnosis, and that collaboration between them is essential in supporting cancer patients [14, 20].

### Interpretation

Concerns classified as CCT immediately after diagnosis were the most prominent, revealing the reality that patients strongly seek accurate information on treatment. The intention to consult hospital pharmacists was particularly high for concerns directly related to cancer drug therapy, such as “Side effects associated with cancer treatment,” “The mechanism of action, efficacy, and effects of anticancer drugs,” and “Medications to alleviate physical discomfort caused by cancer or its treatment”. The fact that hospital pharmacists have a high level of expertise and are able to promptly provide pharmacological interventions, such as prevention and management of side effects and suggestions for supportive care in collaboration with physicians and nurses, is thought to have increased patients'trust in them and led to their need for consultation [21]. The relatively low willingness to consult with community pharmacists may be due to the fact that such pharmacists are generally recognized as a position that delivers and explains medications, and are not easily recalled as a provider of professional consultation regarding cancer pharmacotherapy [14, 22–24].

It is noteworthy that despite the relatively high selection rate of concerns classified as DLCTs, the need for consultation with pharmacists was limited. Concerns such as nutrition management, coping with changes in appearance, and lifestyle precautions are areas where pharmacists can provide assistance from a ‘comprehensive perspective’ [16]. However, they are considered areas that are handled by other healthcare professionals, such as physicians, nurses, and dietitians, and the idea of consulting a pharmacist may not have taken root in patients [25, 26]. In addition, the limited number of pharmacists who can provide appropriate answers and assistance in these areas at the same level as other healthcare professionals may also contribute to the low willingness to consult. Unlike hospital-based healthcare professionals, community pharmacists are able to engage with patients on a daily and ongoing basis, and are in a position to identify minor changes and problems in daily life at an early stage [27]. Therefore, the active involvement of community pharmacists in the area of DLCT may lead to early detection and response to problems and contribute to the maintenance and improvement of patients'quality of life. The acquisition of skills by pharmacy pharmacists to deal with daily life-related support and the raising of awareness that community pharmacists can also provide support will lead to a deepening of expertise that differs from that of hospital pharmacists and contribute to the establishment of a practical role for pharmacists in the community. These findings also reaffirm the importance of pharmacists within the framework of multidisciplinary cancer care. While physicians and nurses play central roles in diagnosis, treatment planning, and psychosocial support, pharmacists are uniquely positioned to contribute through monitoring patients’ medication use and providing medication counseling [28–30]. Community pharmacists are expected to offer feedback to physicians regarding patients’ medication-related information [30]. Close collaboration and effective information sharing between hospital and community pharmacists – and with the broader care team – are essential to delivering coordinated, patient-centered care throughout the cancer care continuum.

Although the selection rate for problems classified as MOCT was relatively low, this may be because these items are not directly related to cancer treatment, making them less prominent in patients’ minds. Nevertheless, more than 40% of respondents expressed a desire to consult with hospital pharmacists, and a similar proportion wished to consult with community pharmacists. This indicates that support was expected from both types of pharmacists. In particular, issues such as drug–drug interactions and the appropriate way to take medications are expected to become increasingly important as cancer treatment continues over time. While hospital pharmacists are responsible for advanced medication management during hospitalization, community pharmacists are expected to provide ongoing support in the outpatient setting, including daily medication management and the provision of drug information [14, 31]. Establishing a support system in the area of MOCT is essential to ensure the safe and continuous implementation of cancer treatment, and this area should be further developed as part of future pharmacy practice.

### Strengths and weaknesses

The strength of this study lies in the fact that it was conducted on a large scale, targeting a broad patient population, without limiting conditions such as cancer type, stage, age, or gender. In particular, the fact that the study focused on the period immediately after cancer diagnosis, when psychological burden is high and concerns are likely to become apparent, is a significant feature of this study. Furthermore, by comparing the consultation needs of hospital pharmacists and community pharmacists using the same index, we were able to clearly capture the differences in their respective professions and patients'expectations. In addition, the fact that the study was not limited to pharmacotherapy support, but also included psychological, social, and economic aspects as evaluation targets, enhances the significance of this study in that it was able to grasp the actual status of pharmacists’ multifaceted role.

However, there are several limitations. First, this study was conducted using a web-based self-administered questionnaire, which may have introduced selection bias favoring individuals with higher IT literacy. Second, we did not collect information on the time elapsed since diagnosis. Therefore, participants’ responses – particularly regarding consultation preferences – may reflect their current impressions rather than those at the time of diagnosis, introducing potential recall bias. Third, information on treatment modalities, for example, oral versus injectable therapies was not collected, limiting our ability to explore differences in support needs by treatment type. Fourth, over 80% of respondents had one of five common solid tumors – breast, colorectal, gastric, prostate, or lung cancer – potentially underrepresenting the concerns of patients with hematologic malignancies or rare cancers. Lastly, the sample size was insufficient to support detailed subgroup analyses, such as by region or age group.

### Further research

To address these limitations, future research should adopt longitudinal designs that consider the time elapsed since diagnosis, enabling clarification of how patients'concerns and support needs evolve across treatment phases. To improve representativeness and reduce selection bias, surveys should combine web-based, paper-based, and in-person approaches, particularly to reach older adults and patients with rare or hematologic cancers. Collecting more detailed clinical information – such as treatment modality and timing since diagnosis – will allow for more nuanced, stratified analyses. Integrating qualitative methods and medical record data can complement self-reported data and help mitigate recall bias. Lastly, expanding sample sizes will support subgroup analyses by demographics, region, and cancer type, contributing to the development of tailored, evidence-based support strategies.

## Conclusions

This study demonstrated that patients have a strong need to consult hospital pharmacists for issues related to cancer pharmacotherapy, while they have greater expectations for community pharmacists regarding medication management and daily life support. These findings align with societal trends, such as the shift to outpatient cancer care and development of integrated community-based care, and may serve as a foundation for clarifying and expanding pharmacists’ professional roles. In the future, building collaborative and continuous support systems involving both hospital and community pharmacists will be essential to meet the diverse needs of patients with cancer. These efforts may contribute to the ongoing development of multidisciplinary cancer care frameworks, where pharmacists collaborate with physicians, nurses, and other healthcare professionals by incorporating diverse clinical perspectives and fulfilling complementary roles to deliver more comprehensive, patient-centered support.

## Data Availability

No datasets were generated or analysed during the current study.

## Abbreviations

CCT: Cancer and Cancer Treatment
CPPC: Cancer Pain and Palliative Care
DLCT: Daily Life during Cancer Treatment
MOCT: Medications Other Than Cancer Treatment
OTC: Over the counter
